# Decreased Peripheral Blood Natural Killer Cell Count in Untreated Juvenile Dermatomyositis Is Associated with Muscle Weakness

**DOI:** 10.3390/ijms25137126

**Published:** 2024-06-28

**Authors:** Amer Khojah, Lauren M. Pachman, Ameera Bukhari, Chi Trinh, Gabrielle Morgan, Surya Pandey, I. Caroline Le Poole, Marisa S. Klein-Gitelman

**Affiliations:** 1 Department of Pediatrics, College of Medicine, Umm Al-Qura University, Makkah 21955, Saudi Arabia; amkhojah@uqu.edu.sa; 2 Division of Pediatric Rheumatology, Ann & Robert H. Lurie Children’s Hospital of Chicago, 225 East Chicago Avenue, Box 50, Chicago, IL 60611, USA; 3 Feinberg School of Medicine, Northwestern University, Chicago, IL 60611, USA; 4 College of Science, Taif University, Taif 21944, Saudi Arabia; 5 Wellesley College, 106 Central St, Wellesley, MA 02481, USA; 6 Robert H. Lurie Comprehensive Cancer Center, Skin Biology and Diseases Resource-Based Center, Chicago, IL 60611, USA

**Keywords:** natural killer cell, juvenile dermatomyositis, disease activity scores, perforin expression

## Abstract

Juvenile Dermatomyositis (JDM) is the most common inflammatory myopathy in pediatrics. This study evaluates the role of Natural Killer (NK) cells in Juvenile Dermatomyositis (JDM) pathophysiology. The study included 133 untreated JDM children with an NK cell count evaluation before treatment. NK cell subsets (CD56^low/dim^ vs. CD 56^bright^) were examined in 9 untreated children. CD56 and perforin were evaluated in situ in six untreated JDM and three orthopedic, pediatric controls. 56% of treatment-naive JDM had reduced circulating NK cell counts, designated “low NK cell”. This low NK group had more active muscle disease compared to the normal NK cell group. The percentage of circulating CD56^low/dim^ NK cells was significantly lower in the NK low group than in controls (0.55% vs. 4.6% *p* < 0.001). Examination of the untreated JDM diagnostic muscle biopsy documented an increased infiltration of CD56 and perforin-positive cells (*p* = 0.023, *p* = 0.038, respectively). Treatment-naive JDM with reduced circulating NK cell counts exhibited more muscle weakness and higher levels of serum muscle enzymes. Muscle biopsies from treatment-naive JDM displayed increased NK cell infiltration, with increased CD56 and perforin-positive cells.

## 1. Introduction

Juvenile Dermatomyositis (JDM) is a systemic autoimmune disease characterized by proximal weakness and skin inflammation [1]. JDM is the most common inflammatory myopathy in pediatrics, with an estimated annual incidence of 2.7–3.4 cases per million [2]. Although the exact pathophysiology of JDM is not clear, the prevailing theory is that the symptoms result from a combination of genetic susceptibility and environmental triggers such as viral infection, exposure to pollution, or ultraviolet light [3,4,5]. Approximately 50% of JDM children have a family history of autoimmune diseases such as systemic lupus erythematosus (SLE) and/or type 1 diabetes, suggesting a shared genetic predisposition [6,7]. Furthermore, JDM children with a family history of SLE exhibit greater interferon-alpha (IFN-α) activity than children without this familial predisposition [6].

The involvement of both the adaptive and the innate immune systems is well documented in JDM pathophysiology [8,9]. For example, myositis-specific antibodies (MSA) are present in more than 50% of JDM patients, and different MSAs are associated with distinct disease phenotypes [1,10]. There is also increased lymphocyte, macrophage, and plasmacytoid dendritic cell infiltration in muscle biopsies of children with JDM [1,11,12,13]. Plasmacytoid dendritic cells are one of the major sources of type 1 interferon (IFN-α and IFN-β) [14], which leads to the increased expression of interferon-regulated genes. Additionally, there is increased production of neopterin, a macrophage-produced metabolite, upon IFN-γ stimulation, which is observed in most untreated JDM patients [15,16,17]. Subsequently, type 1 interferons enhance Natural Killer (NK) cell degranulation and inflammatory cytokine production [9,18,19].

There is growing recognition of the role that NK cell dysregulation plays in the pathophysiology of autoimmune diseases [20,21]. For example, SLE patients have been shown to have decreased peripheral NK cell counts [22]. Furthermore, NK cells display heightened cytotoxicity and a pro-inflammatory profile, which correlates with the downregulation of CD3ξ expression [23]. Similarly, patients with rheumatoid arthritis were found to have decreased peripheral NK cell count [24] with infiltration of the NK cells in the synovial tissue [25,26]. In other conditions, such as recurrent miscarriage, elevated peripheral NK cell counts have been linked with an increased risk of pregnancy loss [27,28].

Decreased circulating NK cell count is associated with orbital myositis disease activity [29]. Furthermore, there is evidence of abnormal NK cell phospholipase Cγ2 signaling and decreased calcium flux in untreated JDM [30]. Despite the increasing evidence, using the NK cell count as a valid indicator of disease activity in JDM is not widely accepted. To attain this goal, this study will examine both the NK cell count in a large cohort of untreated JDM as well as its association with a panel of serologic indicators of JDM disease activity. We also evaluated the reduction of the circulating NK cell subset (CD56^low/dim^ vs. CD56^bight^) in untreated children with JDM as well as its association with muscle tissue infiltration by NK cells.

## 2. Results

One hundred and thirty-three untreated JDM patients are included in this study; 56% of the children with JDM had a low NK cell count (low NK group), and 44% had a normal NK cell count (normal NK groups). The low NK cell group was younger at diagnosis, while no significant differences were observed in terms of sex, race, ethnicity, and myositis-specific antibodies (MSAs) when compared to the normal NK cell group (Table 1).

**Figure 1:**
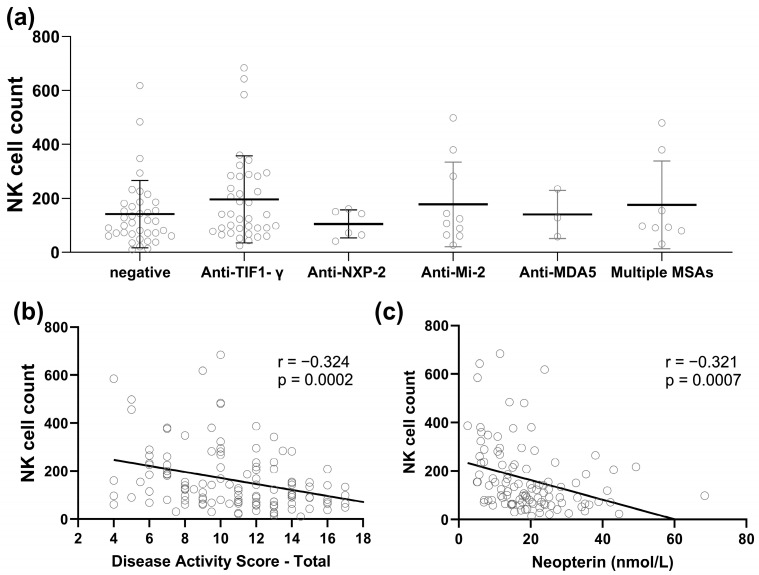
Peripheral blood NK cell count in JDM. (**a**) NK cell counts in children with JDM based on various MSA groups. (**b**) Correlation between NK cell count and total disease activity score. (**c**) Correlation between NK cell count and serum neopterin level.

The low NK cell group had more active disease compared to the normal NK cell group with mean DAS-total 11.6 vs. 9.6 (*p* = 0.001), DAS-muscle 5.8 vs. 3.9 (*p* < 0.001), and neopterin levels 22.1 vs. 15.5 (*p* = 0.003). However, the mean DAS-skin was similar between the low vs. normal NK group (5.8 vs. 5.7, *p* = 0.565) (Table 2). Furthermore, the absolute NK count was negatively associated with DAS-total, DAS-muscle, and neopterin but not DAS-skin (Pearson correlation coefficient of −0.32, −0.33, and −0.32, respectively) (Figure 1). The CMAS was highly influenced by the patient’s age [31]. Of note, the low NK group was significantly younger than the normal NK group. For the CMAS analysis, patients were divided into two groups based on subjects’ age (age ≤4 years and >4 years). The CMAS for children > 4 years old was significantly lower (*p* = 0.01) in the low NK group than in the normal NK group [31], documenting that there was more muscle weakness in the low NK group. Consistent with these findings, the muscle enzymes were higher in the low NK than in the normal NK group as follows: CPK 2730 U/L vs. 465 U/L (*p* = 0.02), AST 146 IU/L vs. 55 IU/L (*p* = 0.003), LDH 491 IU/L vs. 355 IU/L (*p* = 0.007), aldolase 24 U/L vs. 11 U/L (*p* = 0.01).

A review of the different MSA groups documented that the anti-MJ+ children had the lowest mean NK cell count at 127 ± 73 cells/mm^3^, while the Anti-TIF1-γ group has the highest level at 195 ± 160 cells/mm^3^ but related to the small sample size, the difference in NK cell count among the different MSA groups was not statistically significant (Figure 1). With respect to impaired muscle function, the Anti-NXP-2 group has the highest mean DAS-muscle score (6.8 ± 3.4) compared to 4.5 ± 2.6 for the Anti-TIF1-γ group (*p* = 0.001).

**Figure 2:**
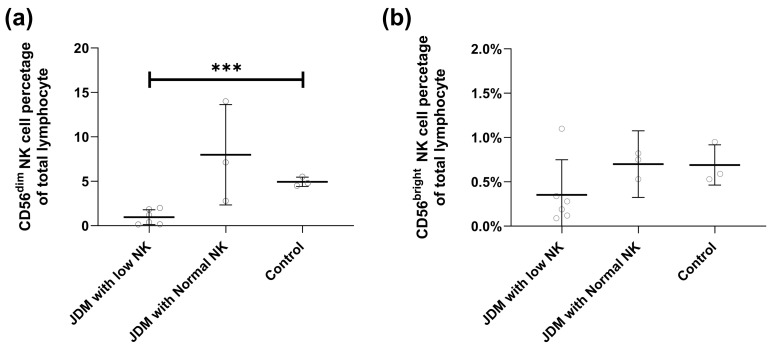
NK cell subpopulation. (**a**) CD56^dim^ NK cell population in JDM vs. control. (**b**) CD56^bright^ NK cell population in JDM vs. control. *** means *p* < 0.001.

To identify the specific NK cell subset (CD56^low/dim^ vs. CD 56^bright^) decreased in the untreated JDM patients, we repeated the flow cytometry to measure the level of CD16 and CD56 expression in six JDM patients with low NK cells, three JDM patients with normal NK, and three healthy pediatric matched controls (Figure 2). Although both subtypes were diminished in the low NK group, the CD56^low/dim^ NK cell population in the untreated children with JDM had a more profound reduction of NK cells compared to their control (0.55% vs. 4.6%, *p* < 0.001) (Figure 2).

**Figure 3:**
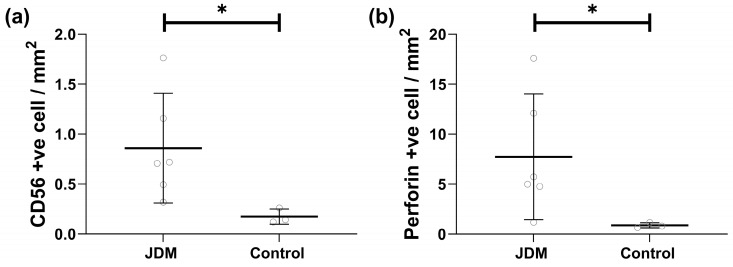
NK cell tissue infiltration. (**a**) Number of the CD56 positive cells in JDM and control muscle biopsies. (**b**) Number of the perforin-positive cells in untreated JDM muscle biopsies. * means *p* < 0.05.

To investigate if the low NK cell counts in JDM reflected NK cell migration into the inflamed tissue, we examined CD56 expression in the muscle biopsy of untreated JDM by immunohistology (Figure 3a). The mean number of CD56-positive cells identified in the muscle biopsy of untreated children with JDM (*n* = 6) was 0.86 ± 0.05 cells/mm^2^, which was significantly higher compared to the control group’s (*n* = 3) mean of 0.17 ± 0.08 cells/mm^2^, *p* = 0.023. Furthermore, the number of perforin-positive cells in an untreated JDM muscle was 8.8-fold greater than in the age and gender-matched control muscle tissue. The mean perforin-positive cell in muscle biopsy of JDM subject was 7.7 ± 6 cells/mm^2^ vs. 0.9 ± 0.3 cells/mm^2^ in controls, *p* = 0.038 (Figure 3b). Of note, these perforin-positive cells colocalized with CD19-positive cells (B cells) 5.4-fold more frequently in JDM tissues than in control samples (* *p* = 0.041) (Supplemental Figure S1).

Next, we evaluated the correlation between the circulating NK cell count and the corresponding serum concentration of CXCL11, a known chemoattractant of T and NK cells that is induced by interferon-gamma and interferon-beta [32]. Serum CXCL11 trended downward in relation to the NK cell percentage with a correlation coefficient of −0.45 (*p* = 0.05) (Supplemental Figure S2a). As expected, there was a strong correlation between CXCL11 and serum neopterin (correlation coefficient of 0.85, *p* < 0.001) (Supplemental Figure S2b). Of note, there was an inverse correlation between serum CXCL10 and NK cell count as well, with a correlation coefficient of −0.46; *(p =* 0.05) (see Supplemental Figure S2c). Additionally, there was a robust correlation between CXCL10 and serum neopterin (correlation coefficient of 0.93, *p* < 0.001) (see Supplemental Figure S2d).

**Figure 4:**
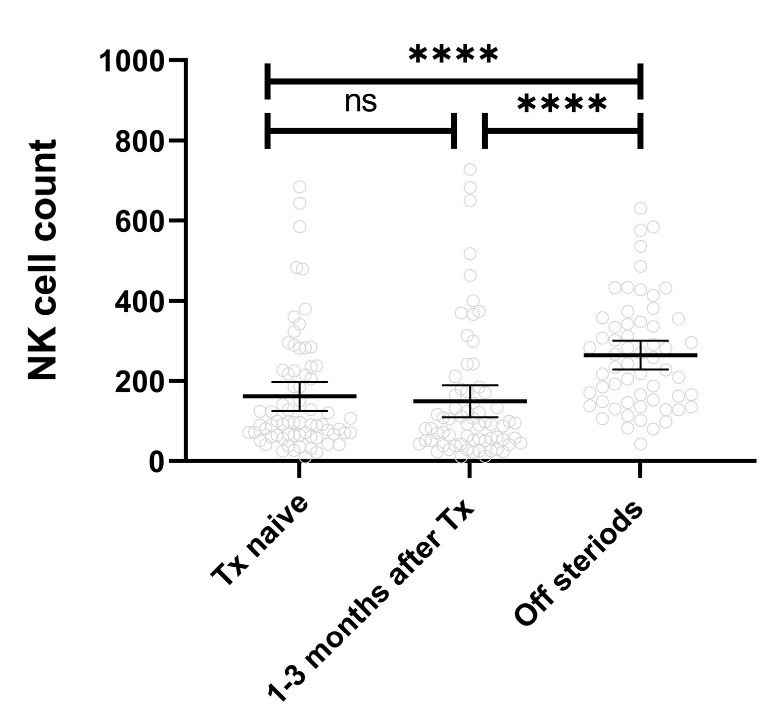
Peripheral blood NK cell count in JDM over time. The mean count of peripheral blood NK cells remained relatively unchanged in treatment-naïve JDM patients compared to the same individuals 1–3 months after starting immunosuppressive drugs. However significant increase in NK cell count was observed after steroid therapy was completed. **** means *p* < 0.0001, ns means not significant.

Lastly, we conducted a longitudinal evaluation of NK cell count in a subset of JDM patients (*n* = 69) with follow-up data over 36 months. There was no significant change in the mean NK cell count between the treatment-naive JDM and the same patients 1–3 months after initiation of immunosuppressive medications. However, a significant increase in NK cell count was observed 1–2 years later (the first sample taken after steroid therapy was stopped) when compared to the same treatment-naive patient samples (mean NK cell count 263.9 ± 138 cells/μL vs. 161.3 ± 150 cells/μL, *p* < 0.0001) (Figure 4).

## 3. Discussion

The presented data support our hypothesis that NK cells play a critical role in the pathophysiology of untreated children with JDM. We had previously documented that NK cell cytotoxicity, but not antibody-dependent cell-mediated killing, is decreased in untreated children with JDM [33]. A recent study revealed that treatment-naïve JDM patients exhibited significant NK cell activation and an increased prevalence of low-ribosome-expressing NK cells [34]. This is associated with reduced NK cell function, which improves with the control of active inflammatory disease [34]. In this study, over 50% of children with JDM had a decreased NK cell count compared to an age-matched reference range (Supplemental Table S1). Untreated JDM with a low NK cell count had more active disease (muscle weakness) and higher muscle enzymes than children in the normal NK cell group. A similar finding has been seen in a case series of JDM [35], orbital myositis patients [29], and SLE patients [22,36]. Of note, a lower circulating NK cell count was also found in adult myositis patients with lung disease than in adult myositis without lung disease [37]. Of interest, the NK cell count correlated inversely with the degree of muscle weakness (measured by DAS-muscle and CMAS) but not skin involvement (evaluated by DAS-skin) or nailfold capillary ERL density. To support this observation, we had previously shown that skin biopsies of untreated children with JDM exhibited a greater number of mast cells compared to healthy controls, but their matching muscle biopsies did not have a similar increase in mast cell infiltration [38]. Furthermore, we had also previously documented that serum neopterin, a macrophage product, tends to be more associated with muscle weakness in JDM than rash [16,17,39].

We next evaluated the NK cell count associated with the different MSA groups [1]. The Anti-NXP-2 positive group had the lowest mean NK cell count, which is consistent with the observation that the Anti-NXP-2 positive group had the highest DAS-muscle score and the highest neopterin levels [16]. In this study, the differences in the mean NK cell counts among different MSA groups had insufficient power for the evaluation. Also, the low NK cell group displayed a significantly higher von Willebrand factor antigen level, which is a biomarker of vasculitis in a subset of JDM patients [40,41], compared with JDM patients whose NK levels were normal.

The NK cells develop from the common lymphocyte progenitors in the bone marrow by gradually downregulating CD34 and upregulating CD56 [42]. The NK cells’ maturation and survival depends on IL-15 or IL-2 [43,44]. Classically, NK cell development consists of five main stages, but only the last two can be detected in the peripheral blood (CD56^low/dim^ and CD 56^bright^ NK cells) [45]. CD56^bright^ NK cells are mostly cytokine-producing cells; they produce cytokines such as IFN γ and GM-CSF and exhibit reduced cytolytic activity [45]. They are typically CD16 negative and can differentiate into CD56^low/dim^ NK cells [46]. CD56^low/dim^ NK cells have more potent cytotoxic activity and less cytokine production. These cells also express CD16 (FcγRIIIA) and killer immunoglobulin-like receptors (KIR) (CD158). In addition, they are capable of secreting perforin and granzyme B upon stimulation. However, the rigid distinction between the two subsets has been called into question by various observations [47]. For example, CD56^bright^ NK cells have demonstrated the ability to kill target cells in certain circumstances [48], and CD56^low/dim^ NK cells can produce IFN-γ [49]. In this study, we found that the reduced NK cell count in children with JDM appears to be associated with a drop in CD56^low/dim^ NK cells. JDM muscle biopsies show increased infiltration of CD56 and perforin-positive cells, which supports enhanced NK cell migration to the inflamed muscle. Tissue-resident NK cells, as observed in this study, usually exhibit the CD56^bright^ phenotype [47]. This has been documented in organs such as the liver and uterus [50,51]. In a study of 108 adult SLE patients, the lupus patients had significantly decreased NK cell counts and cytolytic function in addition to reduced intracellular NK perforin expression [52]. In another report, the NK cell count was low in adults with active lupus, but, unlike our findings, both the NK cell subpopulations (CD56^low/dim^ and the CD 56^bright^ NK cell) were decreased proportionately [22].

Of interest, approximately 10% of the NK cell population in the muscle tissue of JDM colocalized with B cells. The nature of the interaction between NK cells and B cells remains unclear. It is uncertain whether the NK cells actively interact with B cells or if the B cells are drawn to the site of inflammation caused by muscle injury. Nonetheless, this finding emphasizes the intricate interplay between these cell types and underscores the complexity of the disease’s pathophysiology [53]. In our recent study, a B cell subpopulation positive for otoferlin was identified and found to be enriched in treatment-naive JDM compared to control samples [54]. These otoferlin-positive B cells were observed to infiltrate the muscle tissue, adding to the growing body of evidence regarding the involvement of B cells in JDM pathology [54]. Further research is warranted to fully characterize the impact of NK and B cell interactions in JDM pathophysiology.

Although the decreased NK cell count could result from decreased bone marrow production of NK cells, it is more likely due to NK cell migration to inflamed tissue, consistent with our documentation of increased CD56 and perforin-positive cell counts in the untreated JDM muscle biopsy. We postulate that the tissue migration is mediated by the increased production of CXCL10 (IP-10) and CXCL11 (I-TAC) in untreated children with JDM [55]. CXCL10 and CXCL11 bind to CXCR3 (CD183), a chemokine receptor expressed on mature NK cells as well as T cells [56]. CXCR3 may facilitate the migration of NK and T cells to the tissue during infection, especially in a type 1 interferon-rich environment [57,58,59]. It is plausible that muscle inflammation in untreated JDM provides an environment that promotes NK tissue migration, given the high expression of type 1 interferon in the serum and muscle of patients with active disease [15,60]. Furthermore, we recently documented a negative correlation between NK cell count and neopterin levels (r = −0.31, *p* < 0.001) [16]. In a prior study, we showed that neopterin was positively correlated with both CXCL-10 (r2 = 0.88, *p* < 0.0001) and CXCL-11 (r2 = 0.85, *p* < 0.0001). In addition, CXCL-10 production is increased locally in the muscle tissue following muscle damage [61].

**Figure 5:**
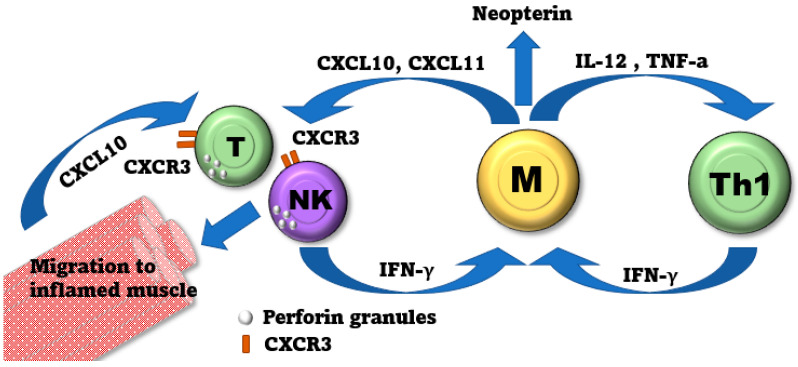
Proposed model of NK cell involvement in JDM. Macrophages and other myeloid cells are stimulated by interferon to produce chemokines CXCL10 (IP-10) and CXCL11 (I-TAC). Additionally, injured muscle cells themselves can also produce CXCL10. These chemokines then bind to a chemokine receptor called CXCR3 (CD183), which is expressed in NK cells and T cells. This binding facilitates the migration of NK cells and T cells to the infected tissue, particularly in an environment rich in type 1 interferon. This migration can potentially lead to further damage to muscle cells in JDM. (M = Macrophages, TH1 = T helper 1 cells, NK = Natural Killer Cells, T = T cells).

Figure 5 shows a proposed model of NK cell involvement in JDM. In summary, the model displays images that show that interferon stimulates myeloid cells to produce CXCL10 and CXCL11 chemokines, which injured muscle cells can also produce. These chemokines bind to the CXCR3 receptor present on NK and T cells, allowing them to migrate to infected tissues, particularly in a type 1 interferon-rich environment. This pathway may provide guidance toward a new therapeutic target for JDM patients who failed conventional therapy.

Many CXCR3 antagonists have been developed to treat autoimmune diseases, but none have yet been FDA-approved [62,63]. Of note, a CXCR3 antagonist reduces the migration of activated T cells to the synovial fluid in a humanized mouse model [64]. Alternatively, an anti-CXCL10 (IP-10) antibody can be used to inhibit inflammatory cell migration to the inflamed muscle [65,66]. We anticipate that understanding the heterogenicity of the inflammatory pathways of JDM will facilitate the development of clinical trials targeting the CXCR3/CXCL10 axis in JDM.

Limitations of this study include the following: (1) the limited number of muscle biopsy analyses hinders the possibility of establishing significant correlations between tissue NK cells and disease activity or peripheral NK cell count; (2) the flow cytometry analysis for NK cell subsets in both JDM patients and controls was performed on frozen samples, which could potentially differ from fresh samples; however, the cell viability was greater than 90% for all samples; (3) the sample size was insufficient to derive a meaningful statistical analysis of the role of NK cells in the presence of specific MSA.

In summary, 56% of untreated JDM children have low peripheral blood NK cell count. Furthermore, those untreated children with JDM and low NK cells have more muscle weakness and higher muscle enzymes than those JDM children in the normal NK cell group. Most of the NK cell count reduction is associated with a lower CD56^low/dim^ population. Additionally, JDM muscle biopsies show increased infiltration of CD56 and perforin-positive cells, which support enhanced NK cell migration to the inflamed muscle. We speculate that targeting NK cells may provide new therapeutic modalities for a subset of JDM patients.

## 4. Materials and Methods

### 4.1. Study Subjects

All patients with JDM seen at Ann & Robert H. Lurie Children’s Hospital are recruited, consented, and given the opportunity to enroll in the Juvenile Myositis (JM) Registry and Biorepository. The data utilized in this study are collected as part of this registry. The Institutional Review Board (IRB) at Ann & Robert H. Lurie Children’s Hospital of Chicago (IRB 2008-13457, 2012-14858, 2010-14306) approved this study. We included all children who met the Bohan and Peter criteria [67,68] for JDM and who had an NK cell count performed before initiating medical therapy. In addition, we excluded subjects with overlap syndrome, such as those with positive anti-PM-Scl, anti-U1 RNP, or anti-U2 RNP antibodies, from the study.

Children with definite JDM (*n* = 133, 75% female, 25% male) were included in the retrospective chart review after they had given their consent for this study. The racial and ethnic background of the study subjects is as follows: White, Non-Hispanic 73% White, Hispanic 19%, African American 3%, Asian 3%, and Others 2%. Age at enrollment (diagnosis) ranged from 2.2–16.9 years with a mean of 6.9 years (±3.7 SD). The mean duration of untreated disease was 8.6 months (±9.9 SD). The distribution of MSAs was: 29.3% Anti-TIF1-γ (anti-P155/140), 8.3% anti-Mi2, 6.4% both anti-Mi2 and Anti-TIF1-γ, 5.3% Anti-NXP-2 (anti-MJ), 2.2% anti-MDA5 (anti-CADM140), and 29.3% MSA negative. 18.2% of the earlier study subjects did not have an up-to-date MSA assessment at the time of this study enrollment, which was prior to the identification of these. To further specify the NK subset involved, more detailed flow cytometry was performed on nine JDM children (6 with low NK cell count and 3 with normal NK cell count) and three age-matched healthy pediatric controls. In addition, tissue staining for CD56 and perforin was performed on diagnostic muscle biopsies from 6 untreated female JDM and three orthopedic, pediatric white female controls (12–14 years old, two had spinal fusion surgery, and one had scoliosis surgery).

### 4.2. Disease Activity Assessment and MSAs

The JDM disease activity evaluation utilized a standardized scoring system at each visit. The Disease Activity Score (DAS)-total consists of 20 points that are categorized into skin symptoms (0–9 points) and muscle symptoms (0–11 points) [69]. Muscle strength was evaluated by The Childhood Myositis Assessment Scale (CMAS) [70,71]. Because both a certified physical therapist is needed to assess the CMAS and patients need to cooperate with the given tasks, some patients did not have CMAS data before therapy, making the sample size too small for a formal evaluation of the CMAS. The CMAS is highly influenced by the patient’s age; healthy children aged four or younger, with a score of 46 instead of 52 obtained by older children [31]. Therefore, the study subjects were divided into two groups based on their age at diagnosis for CMAS analysis. The number of nailfold capillary end row loops (ERL) was evaluated by averaging the number of ERL per mm in the eight digits, excluding thumbs [72,73].

We obtained the following laboratory tests to assess disease activity before treatment: muscle enzymes (creatine phosphokinase [CK], lactate dehydrogenase [LDH], aspartate aminotransferase [AST], and aldolase), erythrocyte sedimentation rate (ESR), von Willebrand factor antigen, and serum neopterin [74]. Serum neopterin was evaluated by a competitive enzyme-linked immunosorbent assay [16]. MSAs were determined via immunoprecipitation and immunodiffusion at the Oklahoma Medical Research Foundation Oklahoma City, OK, USA [75]. CXCL11 and CXCL10 were assessed in 20 JDM patients (13 untreated and 7 post-treatment) by the Meso Scale Discovery^®^ Rockville, MD, USA technique [55].

### 4.3. NK Cells Count by Flow Cytometry

NK cell count was measured by flow cytometry in the clinical immunology lab at the Ann & Robert H. Lurie Children’s Hospital of Chicago. All study subjects had flow cytometry evaluation for CD45 (PerCP-Cy 5.5–labeled CD45, clone 2D1) CD3 (FITC-labeled CD3, clone SK7), CD4 (PE-Cy7–labeled CD4, clone SK3) CD8 (APC-Cy7–labeled CD8, clone SK1), CD16 (PE-labeled CD16, clone B73.1) CD56 (PE-labeled CD56, clone NCAM16.2) and CD19 (APC-labeled CD19, clone SJ25C1) before receiving treatment for JDM. BD Biosciences, San Jose, CA, USA, manufactured all the antibodies for the flow cytometry tests. The normal NK cell count was defined by using the age-specific reference range developed in the Lurie Children’s Hospital Clinical Immunology Lab (Supplemental Table S1). The JDM patients were divided into two groups (normal vs. low) based on their NK cell count. The low NK group was defined as subjects with absolute NK cell count below the age-specific reference range (Supplemental Table S1).

Peripheral blood NK cells can be classified into two groups based on the degree of CD56 expression [45]. CD 56^bright^ NK cells (previously known as immature or stage 4 NK cells) are primarily cytokine-producing cells with low cytolytic activity [45]. They are typically CD16 negative and can differentiate into the CD56^low/dim^ [46]. CD56^low/dim^ NK cells (previously known as mature or stage 5 NK cells) have a more potent cytotoxic activity and lower cytokine production. To identify the specific NK cell subtype (CD56^low/dim^, CD 56^bright^) that is altered in untreated children with JDM, additional flow cytometry was performed on a companion peripheral blood (JDM active, untreated) sample, frozen at −70 °C in the JM Biorepository. The WBCs were stained with CD16 and CD56 and labeled with different fluorochromes. The gating strategy was as follows: (1) dead cells were excluded with fixable cell viability dye (eBioscience San Diego, CA, USA, —eFluor 780); the cell viability was >90% for all the samples; (2) lymphocytes were identified by forward and side scatter in addition to CD45+ve status; (3) CD16 and CD56 antibodies were used to characterize the NK cell subset. Figure 2a shows an example of the NK cells gating strategy.

### 4.4. Immunohistology

For immunoenzymatic staining, 8 μm frozen sections of pediatric muscle tissue biopsies from untreated JDM patients or pediatric orthopedic controls were air-dried, fixed in acetone at 4 °C for 10 min, and stored at −20 °C until use. Tissue sections were stained with the following primary antibodies: AF647-labeled anti-CD56 (Clone: B159—BD Biosciences) or unlabeled anti-human perforin (Clone: B-D4—Biolegend). Alkaline phosphatase-labeled anti-mouse IgGl (Southern Biotech, Birmingham, AL, USA) secondary antibody identified perforin staining. Alkaline phosphatase staining was developed in the presence of Fast Blue BB substrate (Millipore Sigma, St. Louis, MO, USA), followed by the development of horseradish peroxidase by adding either 3,3′-diaminobenzidine tetrahydrochloride substrate (Leica Biosystems, Vista, CA, USA) or 3-Amino-9-Ethylcarbazole (AEC) detection solution (Abcam). Tissues were cover-slipped in glycergel (Dako North America, Inc., Carpinteria, CA, USA). Stained tissues were imaged using a Revolve R4 microscope (Echo, San Diego, CA, USA), and the density of stained cells was enumerated using Adobe Photoshop version 22.1.1 software (Adobe Systems, San Jose, CA, USA).

### 4.5. Statistical Analysis

We divided the study subjects into two groups (low vs. normal NKs) based on the age-appropriate reference range of the absolute NK cell count (Supplemental Table S1). The student’s *t*-test and the chi-square test were used to compare the baseline characteristics and disease activity markers of treatment-naive JDM children with low NK cell count compared with those with a normal NK cell count. We also used Pearson correlation to explore the relationship between various disease activity indicators and NK cell count in untreated JDM subjects. Student’s *t*-test with Welch’s correction was used to assess the difference between NK cell subpopulations and CD56 and perforin expression in the muscle biopsies. We employed IBM SPSS Statistics^®^ version 29.0 and GraphPad Prism^®^ version 9.4.1 to conduct the statistical tests and generate the Figures.

## 5. Conclusions

Fifty-six percent of treatment-naive juvenile dermatomyositis patients exhibit decreased peripheral NK cells. Improvement in NK cell count is observed once the disease progresses into remission. Additionally, muscle biopsies of treatment-naive JDM, albeit with a limited sample size, reveal infiltration of CD56 and perforin-positive cells, indicating increased migration of NK cells to inflamed muscle areas.

## Figures and Tables

**Table 1:** NK cell levels in 133 untreated children with JDM: Demographic characteristics.

	Low NK Cell Group	Normal NK Cell Group	*p*-Value
Number of subjects	75	58	
Age at diagnosis (years), mean ± SD	5.9 ± 3.3	7.6 ± 3.9	**0.009**
Duration of untreated disease (months), mean ± SD	7.5 ± 8.4	10.0 ± 11.4	0.140
Sex, *n* (%)			
Female	59 (79%)	41 (71%)	0.291
Male	16 (21%)	17 (29%)	
Race and Ethnicity, *n* (%)			
White, non-Hispanic	57 (76%)	40 (69%)	0.562
White, Hispanic	13 (17%)	12 (21%)	
African American	2 (3%)	2 (3%)	
Asian	2 (3%)	2 (3%)	
Other	1 (1%)	2 (3%)	
Myositis Specific Antibodies, *n* (%)			
Anti-TIF1-γ	18 (24%)	21 (36%)	0.566
Anti-NXP-2	3 (4%)	4 (7%)	
Anti-Mi2	7 (9%)	4 (7%)	
Anti-MDA5	2 (3%)	1 (2%)	
Multiple MSAs	6 (8%)	3 (5%)	
Negative	26 (34%)	13 (22%)	
Not done	12 (16%)	12 (21%)	

**Table 2:** NK cell levels in 133 children with untreated JDM: Disease activity markers and flow cytometry data.

	Reference Range	Low NK Cell Group	Normal NK Cell Group	*p*-Value
Clinical disease activity indicator				
DAS-total	0	11.6 ± 3.3	9.6 ± 3.1	**<0.001**
DAS-skin	0	5.8 ± 1.6	5.7 ± 1.0	0.565
DAS-muscle	0	5.8 ± 2.8	4.0 ± 2.6	**<0.001**
CMAS for age ≤ 4 years (*n* = 22)	52	26.1 ± 12.6	29.6 ± 10.6	0.491
CMAS for age > 4 years (*n* = 34)	>46	33.6 ± 13.0	44.9 ± 7.4	**0.012**
ERL (#/mm)	>7	5.0 ± 1.8	5.1 ± 1.6	0.710
Laboratory disease activity indicator				
Neopterin (nmol/L)	<10	22.0 ± 10.6	16.1 ± 11.0	**0.005**
ESR (mm/h)	<20	18.6 ± 13.4	15.1 ± 9.7	0.154
von Willebrand factor antigen	Variable *	179.3 ± 81.2	121.2 ± 66.2	**<0.001**
Muscle enzymes				
CK (IU/L)	26–27	2730.2 ± 8049.2	465.7 ± 1244.4	**0.021**
AST (IU/L)	17–96	146.1 ± 239.5	55.0 ± 48.7	**0.003**
LDH (IU/L)	147–463	491.4 ± 353.1	355.0 ± 190.3	**0.007**
Aldolase (U/L)	3.4–8.6	23.5 ± 34.8	11.2 ± 12.7	**0.011**
Flow cytometry				
Total T cells (CD3+)		67.0 ± 9.1	61.7 ± 8.8	**0.001**
T helper cells (CD3+ CD4+)		51.4 ± 45.4	40.4 ± 7.8	0.071
T cytotoxic cells (CD3+ CD8+)		19.8 ± 4.6	19.3 ± 5.3	0.553
B cells (CD19+)		28.2 ± 9.3	29.4 ± 8.4	0.430
NK cells (CD16+/CD56+)		4.4 ± 2.2	8.8 ± 3.2	**<0.001**

* blood type specific: Type B = 57–241%, Type O = 36–157%, Type A = 48–234%, Type AB = 64–238%.

## Data Availability

The data that support the findings of this study are available from the corresponding author upon reasonable request.

## Supplementary Materials

The following supporting information can be downloaded at: https://www.mdpi.com/article/10.3390/ijms25137126/s1.

## Abbreviations

JDM: Juvenile Dermatomyositis, NK cells: natural killer cells, CK: creatine phosphokinase, AST: Aspartate aminotransferase, LDH: Lactate Dehydrogenase, SLE: systemic lupus erythematosus, INF: interferons, MSA: Myositis-specific antibodies, DAS: Disease Activity Score, CMAS: Childhood Myositis Assessment Scale, ERL: capillary end row loops, ESR: erythrocyte sedimentation rate, JM: Juvenile Myositis.
