# Supplementary material for: Decreased Peripheral Blood Natural Killer Cell Count in Untreated Juvenile Dermatomyositis Is Associated with Muscle Weakness
Source: Int J Mol Sci. 2024 Jun 28;25(13):7126. doi: 10.3390/ijms25137126 (PMC11241205; doi:10.3390/ijms25137126)
Supplement: Supplementary file 1 [file ijms-25-07126-s001.zip › ijms-3056399-supplementary.pdf]

Table S1. The reference range of NK cell count and percentage.

	Age (days)	Reference range
NK cell percentage	1 – 3 days old	2 – 8.3 %
	4 – 52 days old	2 – 14 %
	53 – 124 days old	3 – 14 %
	125 – 336 days old	3 – 13 %
	337 – 691 days old	5 –19 %
	692 – 1115 days old	5 – 19 %
	1116 – 1528 days old	4 – 22 %
	>1529 days old	6 – 25 %
NK cells absolute count	1 – 3 days old	64-376
	4-52 days old	60-434
	53-124 days old	194-994
	125 - 336 days old	182-1581
	337 – 691 days old	182-1581
	692 – 1115 days old	164-1171
	1116-1528 days old	121-642
	>1529 days old	138-1027

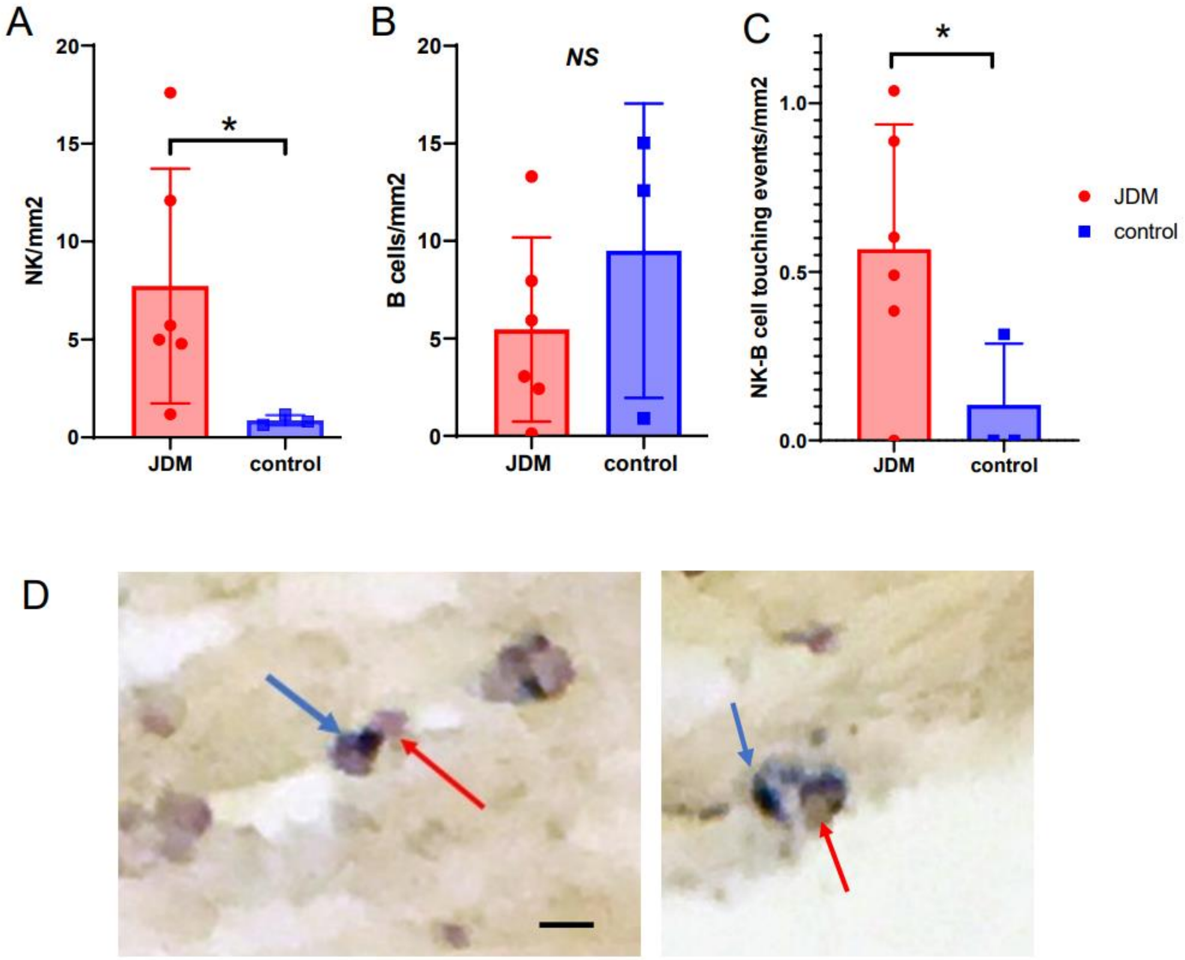

Figure S1. Increased NK cell abundance in JDM muscle tissue. A. NK cells in JDM (n=6) were 8.8-fold more abundant than in adult female muscle tissue (n=3), *p=0.038 as evaluated by perforin staining represented in blue. B. No significant difference was observed in B cell abundance, measured by CD19 staining represented in red. C. Touching events between NK cells (blue arrows) and B cells (red arrows) were 5.4-fold more frequent in JDM tissues than in control samples, *p=0.041. Statistical comparisons were made by 2-tailed, unpaired t-test with Welch's correction. D. Two examples of touching NK cells (blue) and B cells (red)

defined by perforin and CD19 expression, respectively. [JDM sample 10042]. Scale bar for both images: 10 μm.

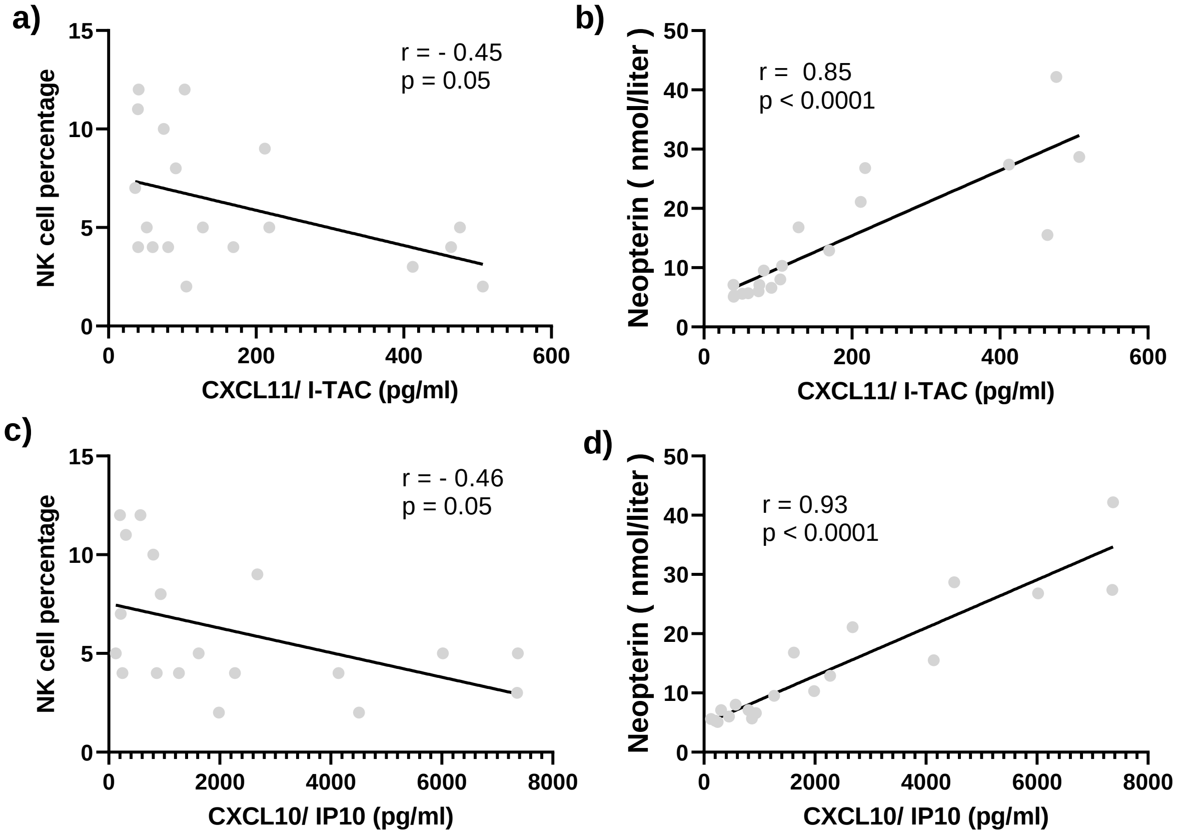

Figure S2. The relationship between the count of NK cells and neopterin compared to the serum levels of CXCL11 and CXCL10.
